# The impact of integrated care on clinical outcomes in patients with alcohol-associated liver disease: Early outcomes from a multidisciplinary clinic

**DOI:** 10.1097/HC9.0000000000000603

**Published:** 2025-02-10

**Authors:** Shreya Sengupta, Akhil Anand, Qijun Yang, Meghan Reagan, Mariah Husted, Austin Minnick, Laura E. Nagy, Srinivasan Dasarathy, Omar T. Sims, Jessica L. Mellinger

**Affiliations:** 1 Department of Gastroenterology and Hepatology, Cleveland Clinic, Cleveland, Ohio, USA; 2 Department of Psychiatry, Cleveland Clinic, Cleveland, Ohio, USA; 3 Section of Biostatistics, Quantitative Health Sciences, Cleveland Clinic, Cleveland, Ohio, USA; 4 Department of Inflammation and Immunity, Cleveland Clinic, Cleveland, Ohio, USA; 5 Department of Gastroenterology, Henry Ford Health System, Detroit, Michigan, USA

**Keywords:** alcohol use disorder, alcohol-associated liver disease, multidisciplinary care, integrated care model, social determinants of health

## Abstract

**Background:**

We analyzed early outcomes regarding the impact of our integrated alcohol-associated liver disease (ALD) clinic on patients with ALD and alcohol use.

**Methods:**

We conducted a retrospective study of patients with ALD who were evaluated in our integrated clinic from May 1, 2022, to December 31, 2023. Primary outcomes included differences in baseline clinical/demographic data between patients who accepted versus declined an appointment and changes in the severity of ALD, alcohol consumption, functional status, hospital utilization, and remission in alcohol use disorder for evaluated patients.

**Results:**

Patients who declined appointments (n=66) had higher median no-show rates (15.0 [8.0,30.0] vs. 8.5 [3.25,15.0], *p*<0.001), social vulnerability index (0.53 [0.26,0.79] vs. 0.38 [0.17,0.63], *p*=0.033), and proportions of cirrhosis (78.8% vs. 59.8%, *p*=0.017) versus evaluated patients. Comparison of baseline to first follow-up visit for evaluated patients (n=102) demonstrated significant reductions in median AST (59.5 [41.75, 89] vs. 44.5 [33.5, 56.25], *p*<0.001), alanine-aminotransferase (33.5 [20,45.25] vs. 26.5 [18.75,33.0], *p*=0.017), total bilirubin (1.6 [0.7,3.3] vs. 1 [0.5,1.9], *p=*0.001), phosphatidylethanol (263 [35, 784] vs. 0 [0, 163], *p*<0.001), MELD-3.0 and Sodium scores for patients with alcohol-associated hepatitis and cirrhosis (16 [11, 18.75] vs. 12 [9, 14], *p*<0.001), 14 [9.25, 17.75] vs. 11 [8.5, 14], *p*<0.001), and Child-Turcotte-Pugh scores for patients with cirrhosis (9 [6, 10.5] vs. 7 [6, 9], *p*<0.001). The proportion of patients with active-severe alcohol use disorder significantly decreased (85.2% vs. 51.9%, *p*<0.001). Additionally, patients had significant reductions in emergency department utilization (incidence rate ratio of 0.64 emergency department visits/month (*p*=0.002) and 0.71 hospital admissions/month (*p*=0.025). However, after considering the false discovery rate, the reduction in hospitalization admissions/month was not statistically significant (False Discovery Rate adjusted *p*=0.056).

**Conclusions:**

Our integrated approach led to reductions in liver injury, degree of liver decompensation, alcohol use, and ED utilization, and remission in AUD in a population of both non-transplant ALD and post-transplant patients.

## Graphical abstract

**Figure:**
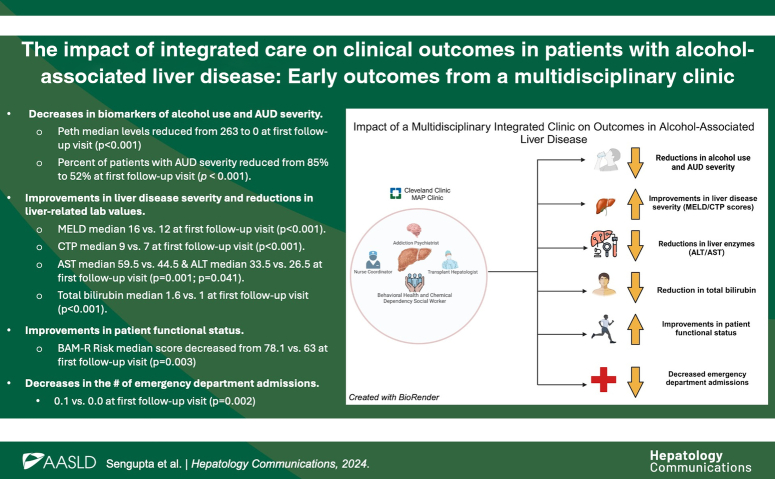

## INTRODUCTION

Alcohol consumption, alcohol use disorder (AUD), and alcohol-associated liver disease (ALD) are significant global causes of morbidity and mortality.^1^,^2^ Worldwide, 2.4 billion people consume alcohol, and the lifetime prevalence of AUD is ~5%–9%.^3^ ALD is one of the gravest consequences of excessive alcohol use and AUD.^4^,^5^ The spectrum of ALD ranges from hepatic steatosis, acute alcohol-associated hepatitis (AH), cirrhosis (AC), and HCC. Alcohol use is the etiology of cirrhosis in approximately 26 million people worldwide, and AC and resultant HCC account for 1% of all global deaths.^3^ Both AUD and ALD pose a significant threat to public health.

Nearly 80% of patients with ALD are diagnosed with a co-existing moderate to severe AUD,^6^ and treatment of AUD is integral in preventing further worsening of ALD. Prior studies have demonstrated that treatment (eg, behavioral and pharmacologic) for AUD can decrease long-term mortality and reduce the risk of development of ALD by 41%–63% and hepatic decompensation by 32%–65%.^5^,^7^,^8^ Despite existing data demonstrating the benefit of AUD treatment for the prevention of ALD and subsequent hepatic decompensation, treatment rates are extremely low.

One of the proposed reasons for low AUD treatment rates in patients with ALD is the lack of widespread integrated care for AUD and ALD. Integrated care models can incorporate hepatology and addiction psychiatry in the care of patients with ALD and allow easier access to necessary addiction and mental health care. Multidisciplinary models can also help overcome clinician-level barriers, such as inadequate addiction training or discomfort with prescribing AUD medications.^9^ Several societies, including the American Association for the Study of Liver Disease and the American College of Gastroenterology, have recommended multidisciplinary and integrated management of ALD and AUD.^10^,^11^ Published data have shown the value of integrated care. A recent meta-analysis demonstrated a significant reduction of >50% in alcohol use and mortality for patients who received integrated care after liver transplant compared to hepatology care alone.^12^ For patients without transplant, a single-center study showed significant improvements in liver function and a decrease in hospital utilization for patients engaged in a multidisciplinary ALD clinic.^13^ Another review demonstrated that integrating psychosocial treatment with comprehensive medical care for patients with AUD and liver disease led to significantly lower rates of alcohol use (32.7%) compared to a control group (75%).^14^ Altogether, multidisciplinary, integrated models have the potential to provide optimal and coordinated care for patients who need simultaneous AUD and ALD treatment.

In May 2022, the Multidisciplinary ALD Program, or Multidisciplinary Alcohol-associated Liver Disease Program (MAP) clinic, was established in Ohio to provide integrated care to patients with ALD and AUD. In this study, we sought to conduct and report several preliminary analyses and patient outcomes from our multidisciplinary model. Specifically, we compared baseline demographic and clinical characteristics between patients who were referred to and seen in the MAP clinic and those who were referred but declined an appointment. For MAP clinic patients, we examined changes between baseline and a first follow-up visit in the severity of liver disease, biomarkers of levels of alcohol use, remission in AUD, and physical functional status; we also assessed changes in patient-reported outcomes (ie, anxiety, depression, insomnia, alcohol use, and overall health) and rates of hospital utilization.

## METHODS

### Study design and setting

We conducted a retrospective study of patients with ALD who were evaluated in Cleveland Clinic’s MAP clinic between May 1, 2022, and December 31, 2023. MAP clinic began operating in May 2022 and was Ohio’s first interdisciplinary clinic for co-management of ALD and AUD. The clinic is staffed by a transplant hepatologist (Shreya Sengupta), addiction psychiatrist (Akhil Anand), behavioral health and chemical dependency social worker (Meghan Reagan), and a hepatology care coordinator nurse is Mariah Husted (MH). Inclusion criteria for this study were the same as inclusion criteria for MAP clinic: a diagnosis of AH or AC, or post-liver transplant for ALD with alcohol use; consumption of alcohol within the past 6 months (even for patients who had transplant); interest in speaking with mental health providers about alcohol use treatment; age over 18 years; and in-state residency. Patients who were undergoing liver transplant evaluation or those registered on the liver transplant waiting list were not eligible for MAP clinic.

MAP clinic was originally held one-half day per week; in January 2023, clinic operations were expanded to two-half days per week. Patients are able to cancel and reschedule appointments on the same day without any penalty, and a waitlist is maintained to provide access to new or existing patients in the event of cancellations or no-shows to the clinic. There are no insurance provider restrictions for patients seeking care in the MAP clinic: patients with public and private insurance, as well as those who self-pay, are eligible.

**FIGURE 1:**
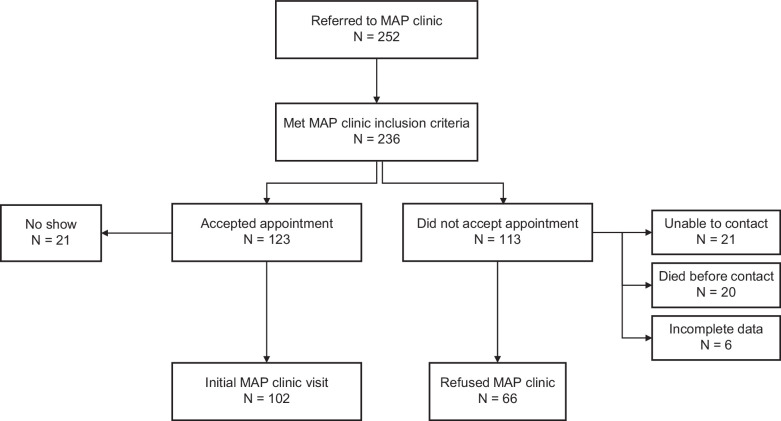
Flowchart of patient selection. Abbreviation: MAP, Multidisciplinary Alcohol-associated Liver Disease Program.

Between May 1, 2022, and December 31, 2023, 252 patients were referred to MAP clinic; 236 patients met MAP inclusion criteria (Figure 1). Of those, 123 patients accepted an appointment, but 21 patients did not attend their appointment and were not included in the analysis. One hundred 2 patients accepted an appointment, were evaluated in MAP clinic, and prospectively followed. Sixty-six patients who declined an appointment and had complete data following the initial referral were included, and their baseline data at the time of referral was compared to baseline data of patients who were referred to and seen in MAP clinic. Patients who declined an appointment were not longitudinally followed.

### Outcomes

Outcomes of interest were (1) demographic and clinical differences between patients with ALD who were referred to and seen in MAP clinic and referred patients who declined an appointment, (2) changes between baseline and a first follow-up visit in the severity of liver disease, biomarkers of levels of alcohol use, the proportion of patients with severe AUD, and physical functional status among MAP clinic patients and (3) changes in rates of hospital utilization and patient-reported outcomes.

### Data collection

Laboratory testing was obtained at every visit for all patients, with interim testing performed as clinically indicated. MELD 3.0 and Sodium (Na) scores were calculated at each visit for patients with AC and AH, and the Child-Turcotte-Pugh (CTP) score was measured at each visit for patients with AC. Every patient underwent alcohol and drug screening at the initial visit and at each follow-up visit with blood phosphatidylethanol, urine ethyl glucuronide, toxicology, and pain panel. Interval testing was performed as clinically indicated. Physical functional assessments were completed using the Liver Frailty Index^15^,^16^ and the Karnofsky Performance Scale score.^17^ The diagnosis of liver disease (AC, AH, or ALD without advanced fibrosis) was determined after clinical evaluation by transplant hepatology (Shreya Sengupta).

AUD, co-existing substance use disorders, and other mental health conditions were diagnosed by psychiatry and social workers (Akhil Anand and Meghan Reagan) using criteria specified in the Diagnostic and Statistical Manual of Mental Disorders Fifth Edition.^18^ History of AUD treatment and medication-assisted treatment (MAT) was determined based on patient interviews and chart review. Patient-reported outcomes related to anxiety, depression, insomnia, alcohol use, and overall health were measured at the initial and at each follow-up visit using generalized anxiety disorder-7,^19^ Patient Health Questionnaire 9,^20^ Insomnia Severity Index,^21^ Brief Addiction Monitor-Revised (BAM-R, Use, Risk, Protective),^22^ and Patient-Reported Outcomes Measurement Information System Global Health 10, which has separate physical (P) and mental (M) scores.^23^ Alcohol use, measured by alcohol biomarkers and patient self-reported data from the BAM-R questionnaire, was studied as a nondichotomous variable, with both abstinence and reduction in overall alcohol use as acceptable end points. We chose the BAM-R questionnaire, a questionnaire that has been validated among patients with substance use disorder and endorsed by addiction medicine societies, which measures alcohol use as well as related risk and recovery and can be self-administered before the visit.^24^

To study hospital utilization for MAP clinic patients, we recorded all emergency department (ED) visits and hospitalizations for the 12 months preceding the initial MAP clinic visit as well as all ED visits and hospitalizations from the time of the initial MAP clinic visit to whichever event occurred first: time of death, discharge from MAP clinic, or the censoring date of December 31, 2023. The Cleveland Clinic Electronic Health Record (EHR) (Epic), as well as Epic Care Everywhere, was queried to find ED visits and hospitalizations outside of the Cleveland Clinic system. ED and hospitalization utilization were calculated as visits per month, and rates were compared pre and post-enrollment in MAP clinic. Demographic data, which included age, sex, race, ethnicity, marital status, insurance payor, and EHR provided an overall “no show” rate, were collected. The EHR provided overall “no show rate” was automatically calculated in each patient record as the total number of any type of visit attended divided by the total number of any type of scheduled visit. For patients who were referred but declined an appointment in the MAP clinic, only baseline data from the time of referral was collected. All research was conducted in accordance with the Declarations of Helsinki and Istanbul.

Social determinants of health variables (eg, related to household conditions, financial strain, and other socioeconomic factors) included the Area Deprivation Index (ADI) and the Social Vulnerability Index (SVI). The ADI is a measure that allows for rankings of neighborhoods by socioeconomic disadvantage in a region of interest and includes information regarding income, education, employment, and housing quality. The ADI, both national percentile and state decile, is calculated for each patient on an annual basis using a social determinants of health questionnaire and is recorded in the EHR for each patient.^25^ Data were collected during visits. The SVI was calculated using a US Census geocoding tool to convert addresses into census tracts according to instructions by the Centers for Disease Control and Prevention.^26^ The SVI is calculated using 16 US census variables split into 4 themes (socioeconomic status, household characteristics, racial and ethnic minority status, and housing types and transportation) to generate a score between 0 and 1. A higher score (closer to 1) indicates increased vulnerability compared to other communities.

With regard to access, the Main Campus is centrally located in areas of higher social vulnerability with public transportation access. MAP clinic is held at the Cleveland Clinic’s Main Campus in Cleveland, OH. This campus is located in Cuyahoga County, which has a national SVI score of 0.73, indicating a medium to high level of vulnerability. At a neighborhood or census tract level, the national SVI for the Cleveland Clinic’s Main Campus is 0.9375, indicating high social vulnerability; the majority of the neighborhoods within an immediate radius of the Main Campus also have high social vulnerability.

All research was conducted in accordance with both the Declarations of Helsinki and Istanbul, all research was approved by the Cleveland Clinic’s Institutional Review Board (IRB approval #23-537), and, and the Cleveland Clinic’s Institutional Review Board approved waiver of written consent. The Strengthening the Reporting of Observational Studies in Epidemiology guidelines were adopted in this study.

### Statistical analysis

Data were described using median and IQR for non-normally distributed continuous variables, and frequency (percentage) for categorical variables. Data at initial visit and first follow-up visit were described to align with the test used for the difference. The Shapiro-Wilk normality test was used to determine the normality of continuous variables. The Pearson Chi-square test and Fisher exact test were used to compare the categorical variables between MAP-seen patients and MAP referred but refused patients. The Wilcoxon rank sum test was used to compare the continuous variables between 2 groups. The paired *t* test and Wilcoxon signed rank test were used to compare the continuous variables between the initial visit and first follow-up visit within MAP-seen patients. The McNemar test and Bhapkar Marginal Homogeneity test were used to compare the categorical variables between 2 visits within MAP-seen patients. The rate of ED visits and hospital admission were compared between pre and post-MAP clinic visits using Poisson regression mixed effects models, accounting for multiple observations per patient, using time group as the predictor, the number of ED visits, hospital admissions as the outcomes, and the follow-up time (months) as an offset. *p*-value was adjusted using a False Discovery Rate of 0.05 to account for multiple comparisons within MAP seen patients. Missing values were excluded from the analyses. Analyses were performed using R software, and a significance level of 0.05 was assumed for all tests.

## RESULTS

### Comparison of MAP patients and those who declined a MAP appointment

Demographic and clinical characteristics were compared between MAP patients and those who declined an appointment (Table 1). Compared to patients who were evaluated in MAP clinic, those who declined had a higher median overall no-show rate (15.0 [8.0, 30.0] vs. 8.5 [3.25, 15.0], *p*<0.001), higher SVI (ie, lived in more vulnerable communities) (0.53 [0.26, 0.79] vs. 0.38 [0.17, 0.63], *p*=0.033), and a higher proportion of AC (78.8% vs. 59.8%, *p*=0.017). Naltrexone was more commonly prescribed for MAT among patients evaluated in the MAP clinic (41.2% vs. 12.3%, *p*=0.001), while patients who declined an appointment were more likely to be prescribed gabapentin (47.7% vs. 27.5%, *p*=0.001). The 2 groups did not differ in other demographics, social determinants of health, history of OLT, and alcohol biomarkers (Table 1). Twenty-seven patients (26.5%) who received liver transplant were evaluated in the MAP clinic; of these patients who underwent liver transplant, 6 (22.2%) had AH of the transplanted liver, and 3 (11.1%) had recurrent cirrhosis in their graft.

**TABLE 1:** Demographics and clinical characteristics of patients eligible for MAP clinic

	All (n=168)	Seen in MAP clinic (n=102)	Declined MAP clinic (n=66)	*p*	N
Demographics					
Age (y)	50.0 [42.0, 58.2]	49.0 [41.2, 59.0]	51.5 [43.0, 57.0]	0.613^a^	168
Sex, n (%)	—	—	—	0.131^b^	168
Female	64 (38.1)	44 (43.1)	20 (30.3)	—	—
Male	104 (61.9)	58 (56.9)	46 (69.7)	—	—
Race, n (%)	—	—	—	0.068^c^	167
White	134 (80.2)	81 (79.4)	53 (81.5)	—	—
Black	22 (13.2)	11 (10.8)	11 (16.9)	—	—
Other	11 (6.59)	10 (9.8)	1 (1.54)	—	—
Ethnicity, n (%)				0.484^c^	163
Hispanic	9 (5.52%)	7 (7.0%)	2 (3.17%)		
Not Hispanic	154 (94.5%)	93 (93.0%)	61 (96.8%)		
Marital status, n (%)	—	—	—	1.000^b^	168
Partnered	63 (37.5)	38 (37.3)	25 (37.9)	—	—
Single	105 (62.5)	64 (62.7)	41 (62.1%)	—	—
Insurance, n (%)	—	—	—	1.000^c^	168
Public	104 (61.9)	63 (61.8)	41 (62.1)	—	—
Private	63 (37.5)	38 (37.3)	25 (37.9)	—	—
Other	1 (0.60)	1 (0.98)	0	—	—
No-show rate (%)	10.0 [4.0, 19.0]	8.5 [3.25, 15.0]	15.0 [8.0, 30.0]	**<0.001** ^a^	168
Social determinants of health					
Social vulnerability index (SVI), state	0.45 [0.20, 0.73]	0.38 [0.17, 0.63]	0.53 [0.26, 0.79]	**0.033** ^a^	162
Area deprivation index (ADI), national percentile	70.0 [47.2, 91.8]	66.0 [41.0, 88.0]	72.0 [52.0, 95.0]	0.115^a^	162
Area deprivation index (ADI), state decile	5.00 [2.00, 9.00]	5.00 [2.00, 8.00]	5.00 [3.00, 9.00]	0.126^a^	162
Liver history					
Liver diagnosis, n (%)				**0.017** ^c^	168
Alcohol-associated hepatitis (AH)	17 (10.1)	15 (14.7)	2 (3.03)	—	—
Alcohol-associated cirrhosis (AC)	113 (67.3)	61 (59.8)	52 (78.8)	—	—
Alcohol-associated liver disease (ALD)	36 (21.4)	24 (23.5)	12 (18.2)	—	—
None	2 (1.19%)	2 (1.96%)	0	—	—
History of OLT, n (%)				0.291^b^	168
Yes	39 (23.2)	27 (26.5)	12 (18.2)	—	—
No	129 (76.8)	75 (73.5)	54 (81.8)	—	—
Alcohol biomarkers					
Phosphatidylethanol (Peth), ng/mL	174 [17, 652]	275 [33, 731]	120 [15, 529]	0.143^a^	135
Alcohol use disorder medications					
Medication-assisted treatment (MAT), n (%)	—	—	—	**0.001** ^b^	167
Other/none	41 (24.6)	24 (23.5)	17 (26.2)	—	—
Naltrexone	50 (29.9)	42 (41.2)	8 (12.3)	—	—
Acamprosate	17 (10.2)	8 (7.84)	9 (13.8)	—	—
Gabapentin	59 (35.3)	28 (27.5)	31 (47.7)	—	—

Bold values are statistically significant.

*Note:* Data were described using median with interquartile range [25th, 75th] and frequency with percentage (%).

^a^ Wilcoxon sum rank test.

^b^ Chi-squared test

^c^ Fisher exact test.

Abbreviations: AC, alcohol-associated cirrhosis; ADI, Area Deprivation Index; AH, alcohol-associated hepatitis; ALD, alcohol-associated liver disease; MAP, Multidisciplinary Alcohol-associated Liver Disease Program; Peth, phosphatidylethanol; SVI, Social Vulnerability Index.

### Baseline psychiatric characteristics of MAP patients

Psychiatric characteristics of patients evaluated in MAP clinic were assessed at baseline (Supplemental Table S1, http://links.lww.com/HC9/B851). Of the 102 patients evaluated in MAP clinic, 84.3% met Diagnostic and statistical manual of mental disorders, fifth edition criteria for severe AUD at their initial visit. The most common co-existing substance use disorder was tobacco use disorder (35.6%). More than three-fourths of patients (76.5%) evaluated in MAP clinic had a co-existing psychiatric diagnosis. The most common co-existing psychiatric diagnoses were major depressive disorder (35.9%), generalized anxiety disorder (17.9%), and post-traumatic stress disorder (15.4%).

### Length of follow-up

The median time between the initial and first follow-up visit was 91.0 days (49.0, 119). The median length of time engaged in MAP clinic (using a censoring date of time of death, discharge from clinic, or December 31, 2023) was 223 days (114, 402) or 7.4 months (3.8, 13.4). Patients were evaluated by every provider (hepatology, addiction psychiatry, and social work) at each visit.

### Laboratory values, physical functional assessments, and AUD diagnosis

For patients evaluated in MAP clinic with more than one visit as of the censoring date (n=54), laboratory data, calculated scores quantifying the severity of liver disease, and physical functional assessments were compared between baseline and first follow-up visit (Table 2). At first follow-up visit, there were significant reductions in median AST (59.5 [41.75, 89] vs. 44.5 [33.5, 56.25], *p*<0.001), ALT (33.5 [20, 45.25] vs. 26.5 [18.75, 33.0], *p*=0.017), total bilirubin (1.6 [0.7, 3.3 vs. 1 [0.5, 1.9], p,0.001), and phosphatidylethanol levels (263 [35, 784] vs. 0 [0, 163], *p*<0.001). Median MELD 3.0 for patients with both AH and AC (16 [11, 18.75, vs. 12 [9, 14], *p*<0.001) and CTP scores for patients with AC (9 [6, 10.5] vs. 7 [6, 9], *p*<0.001) improved from baseline to first follow-up visit; mean MELD-Na scores for patients with both AH and AC also improved (14 [6.15] vs. 11.4 [4.83]). There was a significant improvement in the distribution of Karnofsky scores with higher interquartile ranges at the first follow-up visit compared to baseline (80 [80;90] vs. 80 [70;80], *p*<0.001). Though fewer patients had ascites and encephalopathy at the first follow-up visit, differences were not statistically significant. Similarly, changes in the Liver Frailty Index were not statistically significant. Presence of early or sustained remission from AUD was tracked based on changes in symptomatology using the Diagnostic and statistical manual of mental disorders, fifth edition criteria, and the percentage of patients with an active severe AUD significantly decreased between the initial and first follow-up visit (85.2% vs. 51.9%, *p*<0.001).

**TABLE 2:** Laboratory data, physical functional assessments, AUD diagnosis for MAP clinic patients

	Initial visit	First follow-up visit	*p*	FDR adjusted *p*	N
Laboratory values					
AST	59.5 [41.75, 89]	44.5 [33.5, 56.25]	**<0.001** ^a^	**0.001** ^a^	52
ALT	33.5 [20, 45.25]	26.5 [18.75, 33]	**0.017** ^a^	**0.041** ^a^	52
Albumin	3.7 [3, 4.2]	4 [3.3, 4.4]	0.261^a^	0.329^a^	53
Bilirubin, total	1.6 [0.7, 3.3]	1 [0.5, 1.9]	**<0.001** ^a^	**<0.001** ^a^	53
Creatinine	0.81 [0.65, 0.95]	0.86 [0.69, 1.1]	0.448^a^	0.464^a^	53
Sodium	137 [136, 139]	138 [136, 140]	0.180^a^	0.237^a^	53
PT/INR	1.2 [1.1, 1.33]	1.2 [1.1, 1.3]	0.098^a^	0.142^a^	52
WBC	6.32 [4.98, 7.31]	5.7 [4.78, 6.8]	0.064^a^	0.124^a^	54
Hemoglobin	11.65 [10.32, 13.33]	11.8 [10.3,13.55]	0.948^a^	0.948^a^	54
Platelet count	157 [128, 210]	157 [102, 196]	0.381^a^	0.409^a^	54
Peth	263 [35, 784]	0 [0, 163]	**<0.001** ^a^	**<0.001** ^a^	73
Liver scores					
MELD 3.0 (AH+AC)	16 [11, 18.75]	12 [9, 14]	**<0.001** ^a^	**<0.001** ^a^	38
MELD-Na (AH+AC)	14 (6.15)	11.4 (4.83)	**<0.001** ^b^	**0.002** ^b^	38
CTP (AC)	9 [6, 10.5]	7 [6, 9]	**<0.001** ^a^	**0.001** ^a^	31
Physical exam components of CTP score					
Ascites	—	—	0.087^c^	0.139^c^	32
None	12 (37.5)	17 (53.1)	—	—	—
Slight	10 (31.2)	7 (21.9)	—	—	—
Moderate	10 (31.2)	8 (25.0)	—	—	—
HE	—	—	0.292^c^	0.339^c^	32
None	14 (43.8)	18 (56.2)	—	—	—
Grade 1–2	17 (53.1)	14 (43.8)	—	—	—
Grade 3–4	1 (3.12)	0	—	—	—
Physical function assessments					
LFI (AH + AC)	4.46 (0.6)	4.38 (0.63)	0.305^b^	0.340^b^	24
Karnofsky score	80 [70,80]	80 [80,90]	**<0.001** ^a^	**0.001** ^a^	46
AUD diagnosis					
Severe	46 (85.2%)	28 (51.9%)	**<0.001** ^d^	**0.002** ^d^	54

Bold values are statistically significant.

*Note:* Data at 2 time points were described to align with the test used for the difference using mean with SD, median with interquartile range [25th, 75th], and frequency with percentage (%). Unadjusted and FDR (False Discovery Rate) adjusted *p-*values were reported.

^a^ Wilcoxon signed rank test.

^b^ Paired *t* test.

^c^ Bhapkar Marginal Homogeneity test.

^d^ McNemar test.

Abbreviations: AC, alcohol-associated cirrhosis; AH, alcohol-associated hepatitis; AUD, alcohol use disorder; CTP, Child-Turcotte-Pugh score; INR, international normalized ratio; LFI, Liver Frailty Index; MAP, Multidisciplinary Alcohol-associated Liver Disease Program; Peth, phosphatidylethanol; PT, prothrombin time; WBC, white blood cell count.

### Patient-reported outcomes

Patient-reported outcome measures were compared between baseline and first follow-up visits for MAP patients who had complete data (n=38) (Tables 4A, B). At the first follow-up visit, there were significant improvements in median BAM use (8 [0, 30] vs. 0 [0,6]=0.005) and BAM Risk (78.1 [40.2] vs. 63 [33.3], *p*=0.001) scores (Table 3).

**TABLE 3:** Patient-reported outcome measures for MAP clinic patients

	Initial visit	First follow-up visit	*p*	FDR adjusted *p*	N
BAM-R use	8 [0, 30]	0 [0, 6]	**0.005** ^a^	**0.013** ^a^	38
BAM-R risk	78.1 (40.2)	63 (33.3)	**0.001** ^b^	**0.003** ^b^	38
BAM-R protective	86.9 (27)	95.1 (28.4)	0.083^b^	0.139^b^	35
GAD-7	7 [1, 14.5]	5 [2, 10]	0.091^a^	0.139^a^	43
PHQ-9	7 [4, 15.75]	7.5 [4, 13]	0.274^a^	0.331^a^	46
ISI	14.7 (6.64)	13 (6.23)	0.080^b^	0.139^b^	34
PROMIS (P)	40.4 (10)	41.9 (9.95)	0.158^b^	0.218^b^	38
PROMIS (M)	40.6 (10.7)	43 (9.49)	0.064^b^	0.124^b^	37

Bold values are statistically significant.

*Note:* Data at 2 time points were described to align with the test used for the difference using mean with SD and median with interquartile range [25th, 75th]. Unadjusted and FDR (False Discovery Rate) adjusted *p-*values were reported.

^a^ Wilcoxon signed rank test;

^b^ Paired *t*-test.

Abbreviations: BAM-R, Brief Addiction Monitor—Revised; GAD-7, generalized anxiety disorder 7; ISI, Insomnia Severity Index; MAP, Multidisciplinary Alcohol-associated Liver Disease Program; N, number of patients who had both initial and first follow-up visit data available; PHQ-9, Patient Health Questionnaire 9; PROMIS, Patient-Reported Outcomes Measurement Information System Global Health 10.

### Hospital utilization

The number of ED utilization and hospital admissions per month were compared before and after engagement in MAP clinic. After engagement in the MAP clinic, the Poisson regression mixed effects model demonstrated an incidence rate ratio of 0.64 (95% CIs: 0.48, 0.85; *p*=0.002) for ED visits/month. The incidence rate ratio of 0.71 (95% CIs: 0.53, 0.96; *p*=0.025) for hospital admissions/month was initially significant; however, this change was not statistically significant after considering the false discovery rate (False Discovery Rate adjusted *p*=0.056) (Tables 4A and 4B).

**TABLE 4A:** Hospital utilization for MAP clinic patients.

	Pre-MAP clinic	After initial visit in MAP clinic	*p*	FDR adjusted *p*	n
ED visits/mo	0.1 [0, 0.2]	0 [0, 0.2]	**0.002**	**0.006**	67
Hospital admissions/mo	0.1 [0, 0.2]	0 [0, 0.2]	**0.025**	0.056	67

Bold values are statistically significant.

*Note:* Data were described using median with IQR [25th, 75th]. Unadjusted and FDR (False Discovery Rate) adjusted *p-*values were reported. The Poisson regression using a mixed effects model was used.

Abbreviations: ED, emergency department; MAP, Multidisciplinary Alcohol-associated Liver Disease Program.

**TABLE 4B:** Poisson regression mixed effects model for monthly hospital utilization for MAP clinic patients.

	N	IRR	95% CI	*p*	FDR adjusted *p*
Emergency department	134	—	—	—	—
Pre-MAP	—	—	—	—	—
Post-MAP		0.64	0.48, 0.85	**0.002**	**0.006**
Hospital admissions	134	—	—	—	—
Pre-MAP	—	—	—	—	—
Post-MAP		0.71	0.53, 0.96	**0.025**	0.056

*Note:* Unadjusted and FDR (False Discovery Rate) adjusted *p-*values were reported.

Abbreviations: ED, emergency department; IRR, incidence rate ratio; MAP, Multidisciplinary Alcohol-associated Liver Disease Program; N, number of observations.

### Subgroup analysis of MAP clinic patients previously established with hepatology

We performed a separate subgroup analysis to determine if patients who were previously established with hepatology experienced improvements in outcomes of interest (Supplemental Tables S2–S4C, http://links.lww.com/HC9/B851). Patients who were defined as “established with hepatology” had at least one outpatient visit with a hepatologist from the Main Campus hepatology group in the 12 months prior to their first MAP visit. We compared baseline values with parameters obtained at the first follow-up visit. MAP clinic patients who were previously established with hepatology had significant reductions in AST (57 [37, 92] vs. 43 [31, 56], *p*=0.005), total bilirubin (1.1 [0.5, 2.82] vs. 0.8 [0.43, 1.1], *p*=0.004), MELD 3.0 (15 [11,17] vs. 11 [9, 13.5], *p*=0.001), MELD-Na (12 [8.5, 15] vs. 10 [8, 13], *p*=0.027), and CTP (8.11 [2.52] vs. 7.06 [2.04], *p*=0.005) scores, as well as significant reduction in phosphatidylethanol levels (312.5 [38.75, 837] vs. 27 [0, 209.25], *p*<0.001). After considering the false discovery rate, the only change noted was that the reduction in MELD-Na was not statistically significant (False Discovery Rate adjusted *p*=0.072). MAP clinic patients previously established with hepatology also had significant improvement in performance status as measured by the Karnofsky score (80 [70, 85] vs. 90 [80, 90], *p*=0.001) and a significant reduction in the percentage of patients with a severe AUD at the first follow-up visit (88.6% at baseline vs. 57.1%, *p*=0.015). Significant reductions in BAM-Use (15 [0, 30] vs. 0 [0, 8], *p*=0.011) and BAM-Risk (82.88 [42.82] vs. 62.12 [35.79], *p*=0.002) scores were found. However, differences in number of ED utilization and hospital admissions per month were not statistically significant for MAP clinic patients who were previously established with hepatology (*p*=0.055 and *p*=0.057, respectively).

## DISCUSSION

In this study of characteristics and outcomes of an integrated ALD clinic, we discovered several key findings. First patients who declined an appointment had higher baseline no-show rates, lived in more vulnerable communities as measured by SVI at the state level, and were more likely to have a diagnosis of cirrhosis at the time of referral, which may indicate inequities in access to healthcare. No-show rates are known to be higher in patients with unmet social needs,^27^ with lack of transportation, and forgetting the appointment as the most commonly reported reasons for a missed appointment in a primary care cohort.^28^ Although limited data exists on SVI in patients with liver disease, published data shows that patients who live in areas with high SVI have higher liver related mortality and lower rates of receipt of liver transplant.^29^,^30^ The higher proportion of patients with cirrhosis at the time of referral to specialty care also could suggest potential health inequity in the form of delayed access to diagnosis and appropriate treatment for advanced liver disease. Future studies are needed to determine the association of deprivation indices with outcomes related to ALD, including access to care and clinical outcomes such as severity of disease, rates of return to alcohol use, and overall survival.

Second, patients achieved significant improvements in the severity of liver disease and significant reductions in biomarkers and self-reported levels of alcohol use. In particular, the significant reductions in MELD scores and improvements in median CTP scores achieved by MAP clinic patients have been associated with reduced 90-day mortality, though our study did not prospectively assess 90-day mortality.^31^,^32^ Patients who were seen in the MAP clinic had higher rates of AUD (95.1%) than published data for patients with ALD, which have shown that 75%–80% of patients with ALD have moderate to severe AUD.^6^ We hypothesize that our focus on nontransplant candidates, as well as the inclusion of recipients who underwent transplants with recurrent alcohol use, led to the inclusion of patients with higher addiction severity compared to liver transplant candidates. Despite more severe AUD at baseline, 45% of patients achieved early remission in AUD visits at the time of their first follow-up visit. Patients engaged in MAP clinic not only experienced a reduction in total alcohol consumption but also a significant reduction in risk factors related to alcohol use (measured by BAM-R Risk Score); these findings are related to the principles of alcohol use harm reduction through engagement in MAP clinic.^33^ Although there were no significant changes in the anxiety, depression, and insomnia patient-reported outcome measures between the baseline and first follow-up visit, the generalized anxiety disorder-7 and Insomnia Severity Index scores did improve. We hypothesize that higher patient volumes will allow us to show a significant reduction. It is interesting that the Patient Health Questionnaire 9 scores did not improve between baseline and first follow-up visit, and this requires further evaluation. One hypothesis is that lack of improvement in depression symptoms as measured by the Patient Health Questionnaire 9 could be related to overall medical complexity and continued issues with liver disease, in spite of treatment of addiction and other psychiatric comorbidities. We were unable to perform a subgroup analysis of the individual questionnaires as we only gathered the composite score and not the individual components; however, as we continue to manage this patient cohort and publish our findings, we will ensure more granular data collection regarding these and other questionnaires.

Third, and similar to published data,^13^ engagement in our multidisciplinary model led to a decrease in ED visits (36% reduction) relative to the time prior to engagement in MAP clinic, but the reduction in monthly hospital admissions was not statistically significant, and we plan to recruit and include a larger sample size in an iterative study to potentially overcome statistical issues that are inherent in a small sample size. We noted that all patients, even those who only had one appointment, remained engaged with clinic staff through patient messages and phone calls. Through these nonoffice encounters, patients received urgent and timely medical and psychiatric care as well as medication management, all of which can be common reasons for ED visits. Ultimately, though our study does not show causation, our integrated care model may have led to reduced ED utilization through improved access, including virtual care, and better chronic disease management of both AUD and ALD (similar to what is noted in chronic disease medical home models in primary care^34^). Future research will aim to understand causative factors as well as cost-effectiveness and cost-benefit/utility related to fewer ED visits as a consequence of our integrated model.

Fourth, our subgroup analysis demonstrated that even patients who were followed by a hepatologist prior to receiving care in the MAP clinic benefited from the integrated model. After engaging in the MAP clinic, even patients who were previously established with hepatology experienced significant improvements in markers of liver injury, severity of liver disease, alcohol use, and remission in AUD. This indicates that an integrated approach was associated with improved outcomes for patients with ALD beyond linkage to hepatology care alone.

The reduction in liver injury, achievement of early or sustained remission of AUD, and decreased ED utilization in MAP clinic patients may be related to the many care features of our integrated care model. In addition to a transplant hepatologist, hepatology advanced practice provider, and hepatology care coordinator nurse, our model incorporates addiction specialists: addiction psychiatry, a psychiatry nurse practitioner, and a chemical dependency clinical social worker. Patients are able to receive coordinated hepatology and behavioral health care, pharmacotherapy for AUD, and management of other co-existing psychiatric and substance use disorders in a single multidisciplinary clinic. Hepatology providers (both MD and advanced practice providers) and hepatology-specific care coordination nursing are of utmost importance in appropriately managing a medically sick population who require frequent medical care in between clinic visits; such case management may be responsible for the improved outcomes, but further prospective, controlled research is needed before causation can be established. In the future, we propose to implement equity-focused interventions in MAP clinic for referred patients with high no-show rates and high deprivation indices, which includes additional appointment reminders, transportation assistance to the initial appointment, and ensuring that parking vouchers are available for all attendees to offset the cost of the medical appointment. Our study also sets the stage for future research endeavors to better understand social determinants of health and their impact on health care access and outcomes in patients with ALD, as well as to determine the optimal equity-based intervention in this patient population.

Finally, expanded access to appointments (overbooking, access to individual appointments with appropriate providers, waitlist creation and management) and providers working at the top of their license while also assuming administrative tasks related to the clinic have helped make our model successful. Our clinic also operates in the main psychiatric care hospital within our health system (which also houses the inpatient alcohol and drug withdrawal unit), which has improved access for patients who have already established psychiatry and behavioral health care at this facility. Every member of our clinic contributes to recruitment, scheduling, and patient management. Of particular importance are routine phone calls by care coordination nursing and social work to recruit referred patients, reminder calls before the appointment, and close follow-up via phone call within 1 week after the appointment, which has led to patient retention and engagement with clinic staff and recommendations. As of the censoring date, 93% (95/102) of patients were still being actively followed in clinic. We attribute this high retention rate to the factors discussed above (expanded access to care both during and in between office visits) and our discharge criteria. Once a patient is seen in the integrated ALD clinic, we assume all aspects of their hepatology and psychiatric care, with management of issues ranging from post-transplant immunosuppression to bipolar disorder and depression. Patients are only discharged from the clinic at their request or if a patient moves out of state and cannot come back for appointments. As demonstrated in previous studies regarding integrated care clinics for ALD, a cohesive, co-located team providing flexible, evidence-based, and population-focused care is key to the early successes of our multidisciplinary care model.^35^,^36^

### Limitations and strengths

Limitations of our study include its retrospective nature, single-center experience, missing data for survey and lab variables at follow-up, and the potential for underlying bias with self-referral. The study also lacked a control group when changes in primary and secondary outcomes were assessed. As such, interpretations of our data can only be limited to patients who engaged in MAP clinic. Notwithstanding these limitations, our study assessed biomarkers along with self-reported levels of alcohol use and used validated psychometric scales to assess patient-reported outcomes. Another strength of our study was analysis of both the ADI and SVI, which were developed for different intended uses and contain unique variables (eg, variables related to household conditions and financial strain are part of the ADI).^37^ To date, there are limited data identifying the optimal index for studying SDOH associations with ALD outcomes and, to our knowledge, our study is the first to assess both ADI and SVI in an ALD population. In addition, our study represents the largest cohort of nontransplant patients with ALD and alcohol use evaluated in a multidisciplinary ALD clinic to date and adds to the existing data about the beneficial impacts of an integrated care model for patients with ALD.

## CONCLUSIONS

An integrated care model for patients with ALD, which provides comprehensive hepatology, addiction, psychiatry, and social work care, shows promising early outcomes with regard to improvements in liver disease and AUD. Since multidisciplinary care is considered the gold standard, further research is required to understand the optimal model for concurrently treating AUD and ALD. Our study sets the stage for a larger, multicenter study in which we could examine outcomes for patients with ALD in various integrated care models with appropriate control groups.

## Supplementary Material
