# Supplementary material for: The impact of integrated care on clinical outcomes in patients with alcohol-associated liver disease: Early outcomes from a multidisciplinary clinic
Source: Hepatol Commun. 2025 Feb 10;9(2):e0603. doi: 10.1097/HC9.0000000000000603 (PMC11810017; doi:10.1097/HC9.0000000000000603)
Supplement: Supplementary file 1 [file hc9-9-e0603-s001.docx]

**Table S1. Psychiatric characteristics of MAP clinic patients.**

| Alcohol use disorder (AUD) diagnosis (initial) | N=102 |
| --- | --- |
| Severe | 86 (84.3%) |
|  |  |
| Most frequent co-existing substance use disorder | N = 102 |
| Tobacco use disorder | 36 (35.6%) |
|  |  |
| Most frequent co-existing psychiatric diagnosis | N = 78 |
| Major depressive disorder | 28 (35.9%) |
| Generalized anxiety disorder | 14 (17.9%) |
| Post traumatic stress disorder | 12 (15.4%) |

Data were described using frequency with percentage (%). MAP = Multidisciplinary Alcohol-associated Liver Disease Program.

**Table S2. Laboratory data, physical assessments for patients previously established with hepatology**

| **Variable** | **Initial visit** | **First follow-up visit** | **P-value** | **FDR adjusted p-value** | **N** |
| --- | --- | --- | --- | --- | --- |
| AST | 57 [37, 92] | 43 [31, 56] | **0.005^a^** | **0.020^a^** | 33 |
| ALT | 34 [24, 45] | 26 [19, 34] | 0.057^a^ | 0.122^a^ | 33 |
| ALBUMIN | 3.8 [3.32,4.27] | 4 [3.4,4.35] | 0.965^a^ | 0.965^a^ | 34 |
| BILIRUBIN_TOTAL | 1.1 [0.5, 2.82] | 0.8 [0.43, 1.1] | **0.004^a^** | **0.020^a^** | 34 |
| CREATININE | 0.84 [0.66, 1.05] | 0.86 [0.68, 1.1] | 0.519^a^ | 0.637^a^ | 34 |
| SODIUM | 138 [137, 140] | 139 [136, 140] | 0.570^a^ | 0.637^a^ | 34 |
| PT_INR | 1.2 [1.05, 1.3] | 1.2 [1.1, 1.3] | 0.597^a^ | 0.637^a^ | 31 |
| WBC | 5.99 [4.91, 7.02] | 5.31 [4.67, 6.83] | 0.472^a^ | 0.609^a^ | 33 |
| HEMOGLOBIN | 12 (1.96) | 12.1 (1.79) | 0.579^b^ | 0.637^b^ | 33 |
| PLATELET_COUNT | 145 [127,180] | 156 [98,190] | 0.167^a^ | 0.297^a^ | 33 |
| MELD 3.0 (AH + AC) | 15 [11,17] | 11 [9,13.5] | **0.001^a^** | **0.011^a^** | 23 |
| MELD 3.0 (AC) | 16 [11.2,18.5] | 12 [9.00,13.8] | **0.003^a^** | **0.019^a^** | 19 |
| MELD-Na (AH + AC) | 12 [8.5, 15] | 10 [8,13] | **0.027^a^** | 0.072^a^ | 23 |
| MELD-Na (AC) | 14 [10,15] | 11 [8.25,13.5] | **0.011^a^** | **0.035^a^** | 18 |
| Peth | 312.5 [38.75, 837] | 27 [0, 209.25] | **<0.001^a^** | **<0.001^a^** | 48 |
| LFI (AH + AC) | 4.34 (0.5) | 4.24 (0.41) | 0.343^b^ | 0.523^b^ | 14 |
| LFI (AC) | 4.40 (0.47) | 4.25 (0.43) | 0.228^b^ | 0.384^b^ | 11 |
| CTP (AC) | 8.11 (2.52) | 7.06 (2.04) | **0.005^b^** | **0.020^b^** | 18 |
| Karnofsky | 80 [70, 85] | 90 [80, 90] | **0.001^a^** | **0.011^a^** | 31 |
| Ascites |  |  | 0.589^c^ | 0.637^c^ | 18 |
| None | 10 (55.6%) | 10 (55.6%) |  |  |  |
| Slight | 2 (11.1%) | 3 (16.7%) |  |  |  |
| Moderate | 6 (33.3%) | 5 (27.8%) |  |  |  |
| HE |  |  | 0.371^d^ | 0.540^d^ | 18 |
| None | 8 (44.4%) | 11 (61.1%) |  |  |  |
| Grade 1-2 | 10 (55.6%) | 7 (38.9%) |  |  |  |
| AUD |  |  | **0.015^d^** | **0.044^d^** | 35 |
| Other | 4 (11.4%) | 15 (42.9%) |  |  |  |
| Severe | 31 (88.6%) | 20 (57.1%) |  |  |  |

Data at two time points were described to align with the test used for the difference using mean with standard deviation (SD), median with interquartile range [25^th^,75^th^], and frequency with percentage (%). Unadjusted and FDR (False Discovery Rate) adjusted p-values were reported. a=Wilcoxon signed rank test; b=Paired t-test; c=Bhapkar Marginal Homogeneity test; d=McNemar's test. AST = aspartate aminotransferase. ALT = alanine aminotransferase. PT = prothrombin time. INR = international normalized ratio. WBC = white blood cell count. Peth = Phosphatidylethanol. MELD 3.0 = Model for End Stage Liver Disease. AH = alcohol-associated hepatitis. AC = alcohol-associated cirrhosis. MELD-Na = Model for End Stage Liver Disease – Sodium. CTP = Child-Turcotte-Pugh score. HE = hepatic encephalopathy. LFI = Liver frailty index.

**Table S3. Psychiatric characteristics of patients established with hepatology seen in MAP clinic.**

| **Variable** | **Initial visit** | **First follow-up visit** | **P-value** | **FDR adjusted p-value** | **N** |
| --- | --- | --- | --- | --- | --- |
| BAM Use | 15 [0,30] | 0 [0,8] | **0.011**^a^ | **0.035^a^** | 25 |
| BAM Risk | 82.88 (42.82) | 62.12 (35.79) | **0.002**^b^ | **0.016^b^** | 25 |
| BAM Protective | 90.5 (23) | 93.86 (26.18) | 0.476^b^ | 0.609^b^ | 22 |
| GAD-7 | 9.19 (6.98) | 7.7 (6.01) | 0.153^b^ | 0.288^b^ | 27 |
| PHQ-9 | 9.45 (6.98) | 9.28 (5.75) | 0.865^b^ | 0.893^b^ | 29 |
| ISI | 14.62 (6.41) | 12.21 (6.3) | 0.066^b^ | 0.132^b^ | 24 |
| PROMIS (P) | 40.26 (8.52) | 41.39 (8.94) | 0.412^b^ | 0.573^b^ | 26 |
| PROMIS (M) | 40.4 (10.22) | 42.11 (9.09) | 0.271^b^ | 0.434^b^ | 26 |

Data at two time points were described to align with the test used for the difference using mean with standard deviation (SD) and median with interquartile range [25^th^, 75^th^]. Unadjusted and FDR (False Discovery Rate) adjusted p-values were reported. a=Wilcoxon signed rank test; b=Paired t-test. BAM-R = Brief Addiction Monitor – Revised. GAD-7 = Generalized Anxiety Disorder 7. PHQ-9 = Patient Health Questionnaire 9. ISI = Insomnia Severity Index. PROMIS = Patient-Reported Outcomes Measurement Information System Global Health 10.

**Table S4a. Hospital utilization for patients established with hepatology who were seen in MAP clinic.**

| Variable | Pre-MAP clinic | Post-MAP clinic visit 1 | P-value | FDR adjusted p-value | | N |
| --- | --- | --- | --- | --- | --- | --- |
| ED visits/month | 0.1 [0, 0.2] | 0 [0, 0.2] | 0.055 | 0.122 | | 39 |
| Hospital admissions/month | 0.1 [0, 0.2] | 0 [0, 0.15] | 0.057 | 0.122 | | 39 |
| Data were described using median with interquartile range [25^th^,75^th^]. The Poisson regression using mixed effects model was used. Unadjusted and FDR (False Discovery Rate) adjusted p-values were reported. | | | | |  |  |

**Table S4b. Poisson regression mixed effects model for ED visits per month for patients established with hepatology who were seen in MAP clinic.**

| Characteristic | N | IRR^1^ | 95% CI^1^ | p-value | FDR adjusted p-value |
| --- | --- | --- | --- | --- | --- |
| Time | 78 |  |  |  |  |
| Pre-MAP |  | — | — |  |  |
| Post-MAP |  | 0.69 | 0.47, 1.01 | 0.055 | 0.122 |
| ^1^IRR = Incidence Rate Ratio, CI = Confidence Interval Unadjusted and FDR (False Discovery Rate) adjusted p-values were reported. | | | | |  |

**Table S4c. Poisson regression mixed effects model for hospital admissions/month for patients established with hepatology who were seen in MAP clinic.**

| Characteristic | N | IRR^1^ | 95% CI^1^ | p-value | FDR adjusted p-value |
| --- | --- | --- | --- | --- | --- |
| Time | 78 |  |  |  |  |
| Pre-MAP |  | — | — |  |  |
| Post-MAP |  | 0.68 | 0.46, 1.01 | 0.057 | 0.122 |
| ^1^IRR = Incidence Rate Ratio, CI = Confidence Interval Unadjusted and FDR (False Discovery Rate) adjusted p-values were reported. | | | | |  |
